# Dose-response relationship between the variables of unilateral optogenetic stimulation and transcallosal evoked responses in rat motor cortex

**DOI:** 10.3389/fnins.2022.968839

**Published:** 2022-09-23

**Authors:** Christian Stald Skoven, Leo Tomasevic, Duda Kvitsiani, Bente Pakkenberg, Tim Bjørn Dyrby, Hartwig Roman Siebner

**Affiliations:** ^1^Danish Research Centre for Magnetic Resonance, Centre for Functional and Diagnostic Imaging and Research, Copenhagen University Hospital Amager and Hvidovre, Copenhagen, Denmark; ^2^Center for Functional Integrative Neuroscience, Aarhus University (AU), Aarhus, Denmark; ^3^Department of Molecular Biology and Genetics, Danish Research Institute of Translational Neuroscience, Aarhus University, Aarhus, Denmark; ^4^Research Laboratory for Stereology and Neuroscience, Copenhagen University Hospital Bispebjerg and Frederiksberg, Copenhagen, Denmark; ^5^Department of Clinical Medicine, Faculty of Medical and Health Sciences, University of Copenhagen, Copenhagen, Denmark; ^6^Department of Applied Mathematics and Computer Science, Technical University of Denmark, Kongens Lyngby, Denmark; ^7^Department of Neurology, Copenhagen University Hospital Bispebjerg and Frederiksberg, Copenhagen, Denmark

**Keywords:** rat, optogenetic stimulation, electrophysiology, corpus callosum, transcallosal conduction, primary motor cortex, dose-response

## Abstract

Efficient interhemispheric integration of neural activity between left and right primary motor cortex (M1) is critical for inter-limb motor control. We employed optogenetic stimulation to establish a framework for probing transcallosal M1–M1 interactions in rats. We performed optogenetic stimulation of excitatory neurons in right M1 of male Sprague-Dawley rats. We recorded the transcallosal evoked potential in contralateral left M1 via chronically implanted electrodes. Recordings were performed under anesthesia combination of dexmedetomidine and a low concentration of isoflurane. We systematically varied the stimulation intensity and duration to characterize the relationship between stimulation parameters in right M1 and the characteristics of the evoked intracortical potentials in left M1. Optogenetic stimulation of right M1 consistently evoked a transcallosal response in left M1 with a consistent negative peak (N1) that sometimes was preceded by a smaller positive peak (P1). Higher stimulation intensity or longer stimulation duration gradually increased N1 amplitude and reduced N1 variability across trials. A combination of stimulation intensities of 5–10 mW with stimulus durations of 1–10 ms were generally sufficient to elicit a robust transcallosal response in most animal, with our optic fiber setup. Optogenetically stimulated excitatory neurons in M1 can reliably evoke a transcallosal response in anesthetized rats. Characterizing the relationship between “stimulation dose” and “response magnitude” (i.e., the gain function) of transcallosal M1-to-M1 excitatory connections can be used to optimize the variables of optogenetic stimulation and ensure stimulation efficacy.

## Introduction

The corpus callosum (CC) connects homologous cortical sites in the right and left hemisphere and therefore is a critical structure for interhemispheric integration in the mammalian brain (Olivares et al., 2001; Phillips et al., 2015). This also applies to the premotor and primary sensorimotor cortices. In primates, transcallosal fibers provide an important pathway through which somatosensory information and motor commands from the right and left limbs are integrated with substantial differences in interhemispheric connectivity among cortical areas (Rouiller et al., 1994; Ruddy et al., 2017). Early neurophysiological studies applied cortical electrical stimulation of the cortex in one hemisphere of animals and recorded the “callosal potentials” that were elicited in the homologous part of the opposite hemisphere (Curtis, 1940). Severing the CC at the midline completely abolished the electrically evoked potentials (Curtis, 1940). Augmenting the intensity of electrical stimulation, the recorded peak amplitude in the opposite hemisphere gradually increased without a change in the latency of the peak (Chang, 1953). The peak appeared sharper when the CC was stimulated directly (Chang, 1953), indicating a more synchronized response.

The advent of opto- (Boyden et al., 2005; Aravanis et al., 2007) and pharmacogenetic (Armbruster et al., 2007; Alexander et al., 2009) tools to selectively stimulate a distinct class of brain cells has massively expanded the potential of studying the CC and its function in animals—both *in vitro* (Rock and Apicella, 2015) and *in vivo* (Bocchi et al., 2017; Iordanova et al., 2018; Saiki et al., 2018; Chen et al., 2020). Optogenetic stimulation of the transcallosal somatosensory or motor projections provides a powerful interventional tool to study the directional functional connectivity between such bilateral cortical areas (Iordanova et al., 2018; Saiki et al., 2018; Böhm et al., 2020; Chen et al., 2020). But appropriate use of optogenetic interventions requires detailed knowledge about the stimulus-response relationship between the stimulation variables and the evoked neuronal response in the contralateral hemisphere. Recent optogenetic studies in rodents and non-human primates have shown that the stimulation induced neuronal activity in the brain, reflected by the size of the evoked local field potential (LFP) or the change in axonal firing rates, depends on the stimulation variables, such as the stimulation intensity applied or the duration of laser stimulation (Saiki et al., 2018; Chen et al., 2020; Shewcraft et al., 2020). Although the impact of key variables might have been investigated systematically beforehand in many optogenetic stimulation studies, it often remains unclear why a specific set of stimulation variables was used for optogenetic stimulation in a given study.

Here, we combined optogenetic stimulation with intracortical electrophysiological recordings to characterize functional transcallosal motor-to-motor interactions in anesthetized rats. To establish a robust experimental framework for future studies, we conducted an experiment to identify the optimal stimulation settings for optogenetic stimulation of excitatory neurons in the motor cortex (M1) and their transcallosal projections to the contralateral homologous M1. We hypothesized that the magnitude of the optogenetically evoked intracortical response would gradually increase with stimulation duration and intensity.

## Materials and methods

**FIGURE 1:**
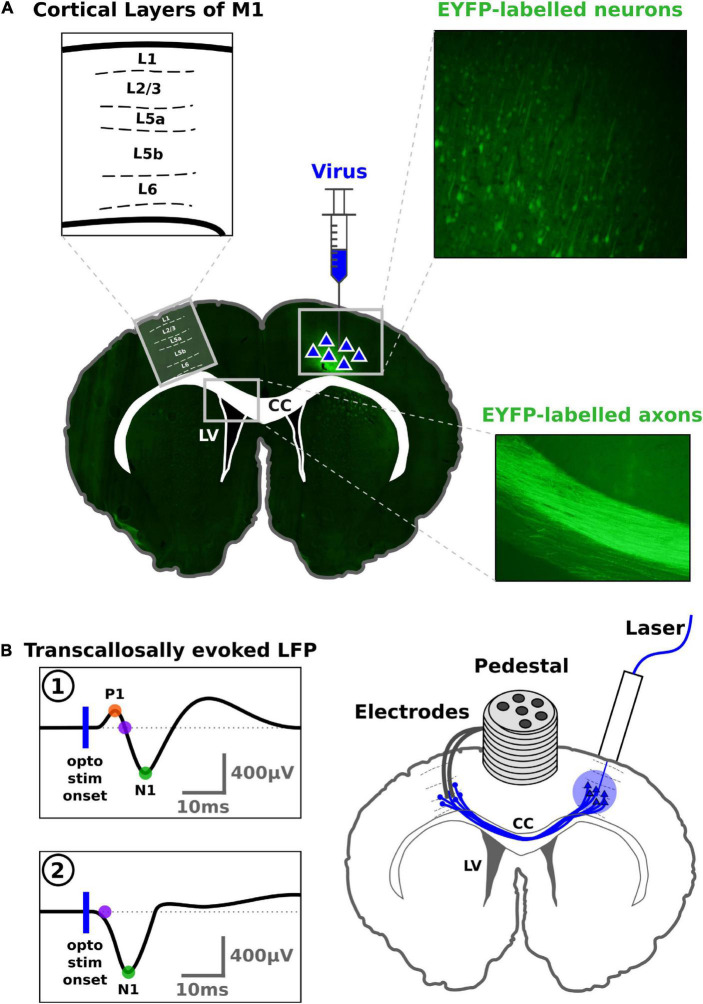
Synopsis of the optogenetic and electrophysiological experimental procedures. The anatomical drawings depict a coronal section (adapted from microscopy images) of the rat brain at approximately +1.00 mm anterior to bregma, according to Paxinos and Watson (1998). The black filled areas indicate the lateral ventricles (LV), while the white central area in the brain corresponds to cerebral white matter, including corpus callosum (CC). **(A)** The viral optogenetic construct was injected in L5 of right M1. As a result, Channelrhodopsin2 (ChR2) and Enhanced Yellow Fluorescent Protein (EYFP) were expressed in the neurons at the injection site and along those axonal projections, projecting to the contralateral hemisphere via the CC. Bright green color in the fluorescence microscopy inserts in CC corresponds to EYFP expressed alongside ChR2. **(B)** An optic fiber was implanted in right M1 for optogenetic excitation of the transcallosal excitatory projection fibers from right to left M1 (depicted as blue lines). A stereo-electrode was implanted in contralateral left M1 to record transcallosal evoked local field potentials (LFP) after optogenetic stimulation of the contralateral cortex. Transcallosal LFP responses displayed two morphologies which are illustrated in the left lower part of the figure. **(1)** The majority of LFP responses showed an initial positive deflection followed by a second negative deflection. The first positive peak (P1) is marked by an orange dot. The first negative peak (N1) is marked by a green dot. The onset latency of N1 (purple dot) was interpolated to the baseline, based on the slope around mid-maximum of the N1 peak. **(2)** Some LFP responses lacked an initial positive component and started directly with a negative deflection (purple dot). The vertical blue line depicts the onset of optogenetic stimulation.

All animal procedures were conducted in accordance with the ARRIVE guidelines, the European Communities Council Directive (2010/63/EU) and were approved by The Animal Experiments Inspectorate (2016-15-0201-00868) in Denmark. Figure 1 illustrates our experimental approach. In young male rats, we stimulated excitatory neurons in the right M1 with a chronically implanted optic fiber and recorded the transcallosal cortical response with an intracortical electrode implanted in the contralateral M1. We systematically investigated the relationship between two key variables of optogenetic stimulation, namely stimulation duration and intensity, and the amplitude of the transcallosal evoked LFP.

### Construction and calibration of the fiber implant

The fiber implants were constructed as follows: a multimode optical fiber (Ø = 55 μm, 0.22 NA, 3 mm protruding; FVP050055065, Polymicro Technologies LLC, CM Scientific) fixed in a ceramic ferrule (L = 6.4 mm, ID: 127 μm, OD: 1.25 mm; MM-FER2007C-1260P, PFP) with cyanoacrylate glue (Loctite Universal). After hardening, every fiber implant was successively polished (30 μm, 6 μm and 3 μm; ThorLabs: LF30D, LF6D, LF3D). Before implantation, the stimulation intensity was calibrated for each optic fiber implant (Sparta et al., 2011). The optic fiber implants were connected (via ADAL1 or ADAL3, Thorlabs) to a custom-made fiber patch cable (5 m, Ø = 50 μm, 0.22 NA, FG050UGA, Thorlabs), which in turn was connected (via ADAFC1) to the fiber-coupled (1 m, Ø = 50 μm, 0.22 NA, FG050UGA, FC/PC) laser source (LaserMate BML447-150FLAM5F, 447 nm, 500 mW). Although many studies use a wavelength for 473 nm for ChR2, we chose a light source with a wavelength of 447 nm. Primarily, because a similar wavelength was also used in the seminal paper for the engineered variant (H134R) used in this study (Nagel et al., 2005)—and as a wavelength of approximately 450 nm has been reported to provide the highest peak and steady-state response (Lin et al., 2009; Lin, 2011). Further, this also gave us more flexibility for combining it with red-shifted opsins in future dual wavelength stimulation/inhibition experiments. The laser was turned on and allowed to stabilize for approximately 15 min (Sparta et al., 2011). Stimulation intensity was measured at a fixed distance and position, by a custom-made 3D-printed (with InnoFil Black Pro1 on Ultimaker 2 + extended) holder,^1^ in relation to the sensor (S121C, ThorLabs) connected to the PowerMeter (PM100A, ThorLabs). The analog dial setting on the laser source was then noted for the different stimulation intensities [0.25, 0.50, 1.00, 2.00, 5.00, 10.00] mW (from approximately 105 to 4,209 mW/mm^2^) for each fiber implant.

### Surgical procedures

Twenty-one young male Sprague-Dawley rats (NTac:SD-M; 3 weeks old; Taconic, Lille Skensved, Denmark) were received and acclimatized at our animal facility for 1 week before they underwent stereotaxic surgery under Isoflurane anesthesia (5% induction, 1.5–3% maintenance; O_2_: 0.5–1.0 L/min; Atm.Air.: 0.5–0.0 L/min). Fur on the head was shaved and cleaned three times with 70% ethanol and 0.5% chlorhexidine in 85% ethanol. Pre-operatively the animals were administered Buprenorphine (Bupaq; 0.3 mg/mL; 0.03 mg/kg), Carprofen (Norodyl/Rimadyl; 50 mg/mL; 5 mg/kg), and sterile saline (0.9%; 5 mL/kg). A mixture of Lidocaine (5 mg/kg, 10 mg/mL) and Bupivacaine (1 mg/kg; 5 mg/mL) was injected subcutaneously on the scalp, 10 min. before incision. The rectal temperature was monitored and maintained at 37.5°C on a heat pad (Harvard Apparatus Homeothermic Monitoring System). Heart rate (HR) and oxygen saturation SpO_2_ was monitored during surgery (Nonin 2500A VET) and used to adjust isoflurane level and (O_2_:Atm.Air)-ratio. Viral inoculation as well as chronic implantation of the optic fiber, electrodes and pedestal were carried out in the same surgical session to reduce the number of surgeries per animal. All stereotaxic coordinates were normalized to a Bregma-Lambda distance of 9.00 mm to correct for the smaller brain size of the 4 weeks old animals (Paxinos and Watson, 1998).

Craniotomies were made above M1 using the following stereotactic coordinates relative to bregma: Anterior-Posterior (AP): +1.0 mm; Medial-Lateral (ML): +2.5 mm (right M1) and -2.5 mm (left M1), and dura was punctured. In right M1, 1 μL of pAAV-CaMKIIa-hChR2(H134R)-EYFP (titer: 4.1–4.6 × 10^12^ virus molecules/mL; UNC Vector Core) was injected at 0.2 μL/min, using a Dorsal-Ventral (DV) penetration depth of -1.00 mm relative to dura to reach L5 of M1.

A custom-made and calibrated optic fiber implant (see previous section) was implanted at the same position. In left M1, a twisted electrode pair made of stainless steel (Plastics1; E363/3-2TW; two wires, Ø = 127 μm each) was implanted at a penetration depth of -1.00 mm DV, relative to dura. Two screw electrodes (Plastics1; E363/20/2.4) were inserted into craniotomies for reference (AP: +8 mm; ML: +0.75 mm) and ground (AP: -3.00 mm; ML: -3.00 mm). All electrodes were connected to a six-channel plastic pedestal connector (Plastics1; MS363). Optic fiber, depth, and screw electrodes as well as the pedestal was cemented to the skull using dental acrylic cement (3M RelyX Unicem and GC Fuji Plus; or Panavia V5: Clearfil; Tooth Primer; Paste). Later animals had the implant embedded within a custom-made and 3D-printed implant protector (see footnote 1) with a removable lid, to reduce damage to and dust on implants. The wound was sutured (Ethicon Vicryl, 5-0 Vicryl, FS3, 16 mm) and another volume of saline (5 mL/kg) was injected subcutaneously to accelerate rehydration. The animals were set to wake up on the heating pad with continuous flow of (O_2_:Atm.Air) in the gas mask, without isoflurane—and subsequently put back in a clean cage, with free access to water as well as solid and softened hydrated food.

### Postoperative treatment

Animals were allowed to recuperate one week in quarantine, with postoperative analgesic treatment of Carprofen (Rimadyl/Norodyl; 50 mg/mL; 5 mg/kg) and antibiotic treatment of Enrofloxacin (Baytril; 50 mg/mL; 10 mg/kg), once daily for 5 days or as needed. Hereafter animals were returned to similar housing, bedding, and enrichment in the animal housing facility. Animals were not included in experiments until 4 weeks after surgery. The rat pedestal protector improved wound healing and allowed us to house two animals together hereafter.

### Measurement of the optogenetically evoked transcallosal local field potentials

Optogenetic stimulation and electrophysiological measurements were carried out four to seventeen weeks after surgery.

#### Anesthesia during optogenetic stimulation

Animals were anesthetized with Isoflurane (5%; 1 L/min @ 50% O_2_ + 50% Atm.Air.), which was downregulated to 2–3% for insertion of tail vein catheter. The catheter was flushed with heparinized sterile isotonic saline (Leo Pharma; 5000 IE/a.e./mL; 0.33 mL Heparine per 100 mL Saline) to avoid clotting. Heart rate (HR) and SpO_2_ was monitorized (Nonin 2500A VET) and logged/visualized with custom-made scripts.^2^ A small bolus (“fast infusion”: ∼0.1 mL/10 s) of Dexmedetomidine (Dexdomitor; Orion Pharma; 0.1 mg/mL) was administered (Harvard Apparatus Pump 11, Pico Plus Elite) until a steep decrease in HR (∼20%; e.g., 375 to 300 bpm within 15 s) was observed. Regular infusion was continued (0.05 mg/kg/h. for the first hour; 0.15 mg/kg/h. hereafter) in combination with a downregulated isoflurane level (0.5%; 1 L/min) (Pawela et al., 2009; Magnuson et al., 2014).

#### Electrophysiological recording and optical stimulation setup

Animals were then placed in a Faraday cage (Campden Instruments; CI.80604E-SAC-H1). Body temperature was monitored and stabilized at ∼37.5°C on a heat pad (Harvard Apparatus Homeothermic Monitoring System) throughout the experiment. The fiber optic patch cable (L = 5 m, Ø = 50 μm, Thorlabs) was connected to the ceramic ferrule embedded in the cranial cement with a ceramic ferrule sleeve connector (Thorlabs ADAL1). The electrode pedestal was connected to a 4-channel differential bio-amplifier (Warner Electronics DP-304A) through a connector cable (Plastics1; 363-000) custom adapted to BNC-plugs through a Cat7 ethernet cable with four twisted pairs. Each of the active channels were connected to individual wires in each pair, and the shared reference electrode connected to the other wire in each pair. The ground electrode was connected to the electric shielding in the custom-adapted cable. Gain was set to ×1,000 for the first animals (*N* = 4), but reduced to ×100 in subsequent recordings to avoid clipping of the signal. Hardware filters were set to bandpass the signal between 0.1 and 50 kHz. The amplified signal was recorded and digitized at 30 kHz on a Lenovo T460 laptop (Ubuntu Linux 18.04) using an Open-Ephys acquisition board and software (v.4.5.0) (Siegle et al., 2017).

Once the animals were stable (HR, SpO_2_ ≥ 98%, T = 37.5 ± 1°C) they were exposed to laser stimulation (447 nm) according to a pseudo-randomized parameter mapping paradigm as follows below. The laser and optic fiber patch cables were the same as used for previous calibration of the optic fiber implants. The stimulation variables of optogenetic stimulation were controlled by an open-source Pulse Generator (PulsePal rev.2, firmware v.2.0.1, Sanworks) (Sanders and Kepecs, 2014) with custom-made python scripts (code available at https://git.drcmr.dk/cskoven/PulsePal).

### Optogenetic stimulation paradigm

We applied optogenetic stimulation to right M1 using different combinations of stimulation durations and stimulation intensity. We recorded the cortical response in left M1, evoked by optogenetic stimulation in right M1 and propagated directly through corpus callosum. In total, seven different stimulation durations (0.1–10.0 ms) and six different stimulation intensities (0.25–10.0 mW) were combined, resulting in 42 duration-intensity settings. For each duration-intensity combination, we applied 150 stimuli, recording a total of 6,300 optogenetically evoked LFPs in the left M1. After the initial four experiments, we made some adjustments to improve signal-to-noise ratio. In the first four animals, we tested each of the six levels of stimulation intensities (0.25, 0.50, 1.00, 2.00, 5.00, 10.00 mW) in one block (gain: ×1,000). At each stimulation intensity, we randomly intermingled blocks of 50 trials at three different stimulus durations. Hence, stimulations at a given intensity were carried out in one train and were thus placed relatively fixed in the anesthetic paradigm. A few trials (<8 trials per condition) had to be discarded due to technical reasons. To spread out the various stimulation duration–intensity combinations throughout the experiment, we randomly intermingled blocks of multiple combination in all subsequent experiments. For each stimulation intensity, three trains were carried out having 2 blocks of 25 trials for each of the seven different stimulation durations, with these blocks being randomly intermingled (gain: ×100). Initially, trial duration was fixed to 1,000 ms which led to an accumulation of 50 Hz line noise during blocks of measurements. We therefore increased the trial duration slightly (to π/3 ≈1.0472 s) to secure a trial-by-trial shift in phase for the underlying 50 Hz line noise in later experiments. In summary, our adjustments reduced the effects of line noise and potential impact of physiological variables as a function of the anesthetic depth. Since overall stimulation conditions were kept constant, data acquired before and after these adjustments were pooled. The change in signal amplification was handled during signal processing. Total recording duration was approximately 3 h.

### Data analysis

When exposing the right M1 to laser stimulation, the typical transcallosal response evoked in left M1 entailed a small positive peak (P1) and a subsequent and more prominent negative response (N1), corresponding to response pattern “1” in Figure 1B. In some animals, the P1 peak was either absent or too small for automatic detection, corresponding to response pattern “2” in Figure 1B. Since the N1 peak was robustly detected in almost all animals, our analyses focused mainly on the N1. Data processing and analysis was performed using custom-made scripts programmed in Python3^3^ and open-source reading tools for electrophysiological data.^4^ Briefly, the processing included notch filtering at 50 Hz and 2nd order Butterworth band-pass filtering between 3 and 300 Hz. Sessions recorded with a gain factor of ×100 instead of ×1,000, were multiplied by a factor 10 as compensation. Single trials included all data points recorded 900 ms before and after the onset of optogenetic stimulation. The recorded signal from the two channels for the stereotrode were averaged for each individual trial. Trials were further baseline corrected using the mean value of the data points in the 10 ms period before laser stimulation onset. Trials were grouped according to duration-intensity condition (42 conditions: 7 stimulation durations and 6 stimulation intensities).

#### Peak detection

For each condition, trials were averaged. The first positive peak (P1) and negative peak (N1) of the LFP response in M1 were automatically detected, if their amplitude extended beyond the threshold of 1 standard deviation based on the 100 ms preceding the stimulus onset. Peak detection was restricted to a time window of 1–9 ms after stimulation onset for P1 and 5–20 ms for N1. Automatic peak detections were verified by CSS, to eliminate possible spurious detections. Peak onset was interpolated (Kaur et al., 2004; Makarov et al., 2008), and defined by the intersection of a first order regression line on the peak slope, between 45 and 55% of the peak maximum, and the baseline. Color-coded group grids of the evoked transcallosal response in M1 were created to describe the detectability (Figures 3A,B), variability (Figures 4A,B) and amplitude (Figure 4C) of the transcallosal M1 response evoked by optogenetic stimulation of the contralateral M1.

**FIGURE 2:**
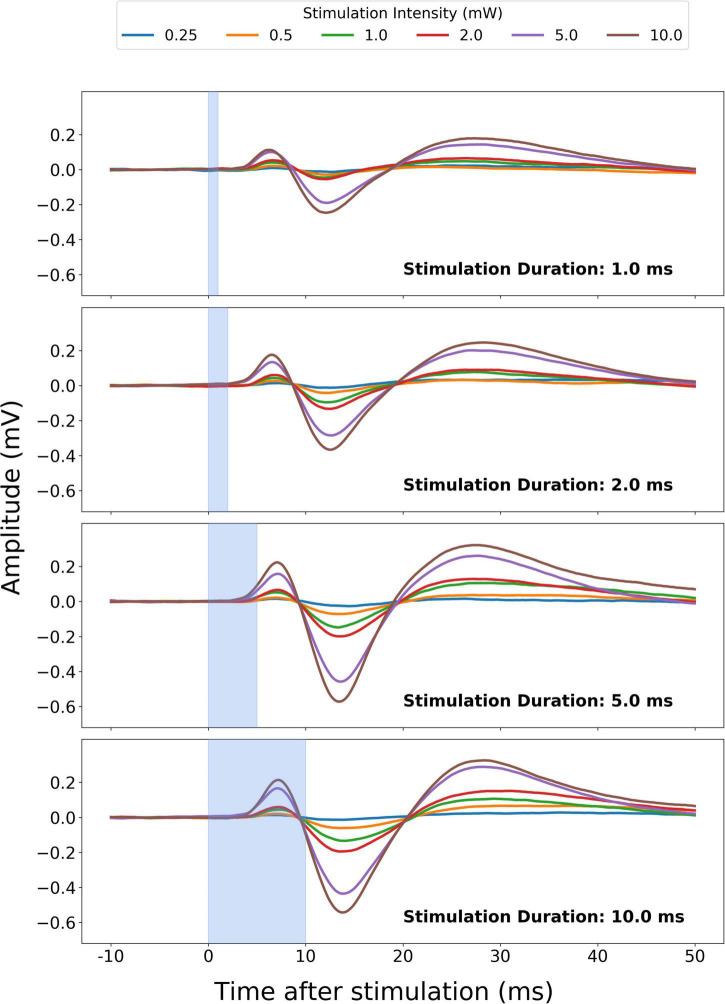
Transcallosal neural responses in left motor cortex (M1) evoked by contralateral optogenetic stimulation of right M1. Optogenetic excitation of transcallosal projection fibers to contralateral left M1 gave rise to transcallosal evoked potentials in left M1. The mean evoked local field potential (LFP) recorded from one animal (rat27.2, 141 days old, 111 days after surgery), showing an early positive component (P1) and a subsequent negative component (N1). The four panels depict responses evoked with optogenetic stimulation at four different stimulation durations out of seven different durations in total. The different colors correspond to different stimulation intensity levels.

**FIGURE 3:**
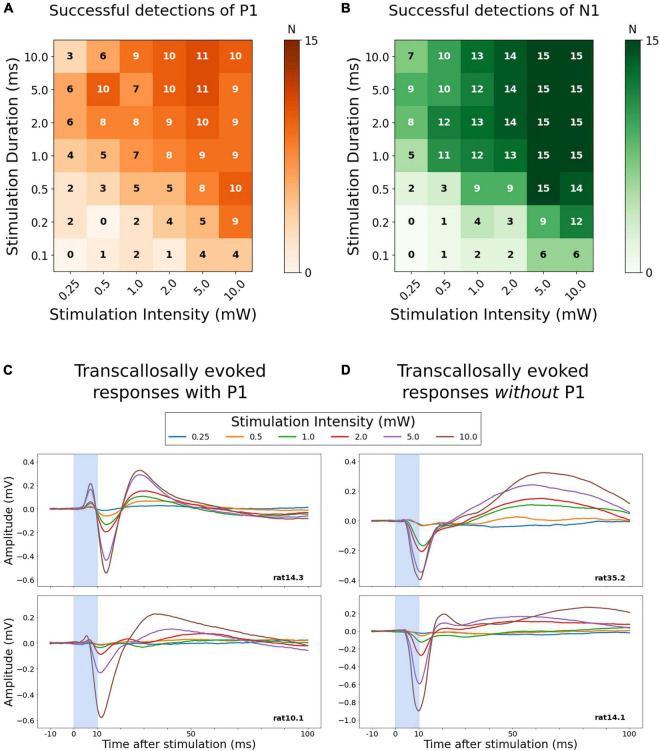
Detectability of a transcallosal P1- or N1-peak response depending on stimulation intensity and duration. **(A,B)** The upper panels show color-coded grids indicating the relative frequency of successful P1 peak detection (orange) or N1 peak detection (green) in left M1 after optogenetic stimulation of right M1. Each grid cell corresponds to one of the 42 combinations of stimulation intensity (x-axis) and duration (y-axis). The numbers plotted inside each cell of the grid indicate the absolute number of animals showing a detectable peak. The lower panels depict averaged samples traces of the transcallosal LFP of four animals. Two animals show a P1-peak **(C)**, while a P1 peak is absent in the other two animals **(D)**. Stimulation duration was fixed (10 ms). Stimulation intensities are indicated by color coding.

**FIGURE 4:**
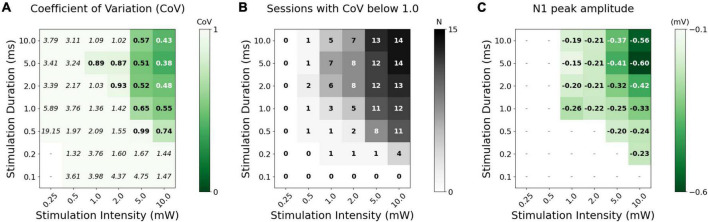
Stimulus-response characteristics of the N1-component of the transcallosal evoked LFP in left M1. **(A)** Median of robustness of the N1 response across animals, determined as coefficient of variation (CoV; SD/mean) of peak N1 amplitude for individual recorded trials. We selected a CoV-threshold for robustness of the N1 peak of 1.0 (values below threshold are marked in bold). **(B)** Number of animal sessions with robust conditions (CoV < 1.0). **(C)** Median N1 peak amplitude (mV) of robust conditions (CoV < 1.0) with N ≥ 20% of the animal sessions. Median values are only shown where there is ≥20% animal sessions for a given condition.

#### Statistics

The relationship between the stimulation parameters (intensity and duration) and the N1 peak amplitudes were investigated statistically using a linear mixed effects model (LMEM; statsmodels, Python3). Stimulation intensity and duration were set as fixed effects and subject ID as random effect. As the output metrics were not normally distributed, the input labels were permutated 10,000 times within subject (all conditions for the interaction effect, and only intensity or duration parameters for the respective main effect) producing z-static for all permutations. *P*-values for the main and interaction effects were calculated as the number z-statistics as or more extreme than the z-statistic of the unpermuted data divided by number of permutations + 1 (Anderson and Braak, 2003; Ojala and Garriga, 2010). All z-scores were normalized with respect to distribution mean and standard deviation. Alpha-level was set to 0.05, resulting in two-sided tails of 0.025 for statistical test of significance of z-scores from LMEMs.

### Euthanization

After the experiments, the animals were anesthetized with a mixture of Hypnorm^®^ (Fentanyl citrate 0.315 mg/mL, Fluanisone 10 mg/mL), Dormicum^®^ (Midazolam, 5 mg/mL), and sterile water in a 1:1:2 ratio (3 mL/kg). The animals were then euthanized by transcardial perfusion of 0.1M potassium phosphate buffered saline (KPBS) followed by 4% paraformaldehyde (PFA; CellPath, Newtown, Powys, UK). The extracted brains were kept in 4% PFA until further use.

### Structural magnetic resonance imaging

A random subset of the brains (*N* = 5) was or obtaining high-resolution structural images, for validation of the electrode and optic fiber position. At least 14 days before the *post mortem* brains were scanned, they were transferred to a vial with fresh 0.1 M KPBS (Shepherd et al., 2009). Immediately before scanning, the brain was wrapped in cloth-tissue (Non-woven swabs, Selefa; OneMed, Danderyd, Sweden), and put in a double-lined plastic bag filled with 0.1M KPBS (Dyrby et al., 2011). The brains were individually scanned using a 7 T preclinical Bruker scanner (Bruker BioSpec 70/20 USR, Bruker Biospin, Ettlingen, Germany) with Paravision 6.0.1-software. A “true” fast imaging with steady-state free precession T2-weighted sequence was acquired with the following parameters. Repetition Time: 2500 ms; Echo Time: 5.1 ms; matrix size: 256 × 256 × 128; Field of View (FOV): 23.04 × 23.04 × 11.52 mm^3^; Image resolution: 90 × 90 × 90 μm^3^; Flip angle: 30°; Averages: 40. Total acquisition time was approximately 2 h. The images were used for validation of location and manual measurements of implantation depths.

### Histology

For histological investigations, some of the brains were sliced with vibratome (LEICA VT1000s) to obtain 40 μm coronal brain sections. For immediate inspection of EYFP-expression, the slices around the injection site were mounted on SuperFrost^®^ slides (Menzel Gläser; ThermoFisher Scientific, Braunschweig, Germany) with Fluoroshield (w/DAPI, #F6057, SigmaAldrich) and cover-slipped (size #0, Menzel Gläser). For validation, some sections underwent immunohistochemical staining procedures. The sections were washed thrice in KPBS and preincubated in KPBS containing 5% goat serum, 1% bovine serum albumin (BSA) and 0.3% Triton X-100 for 30 min. Following, they were incubated with primary antibodies for vGlut2 (polyclonal guinea pig, anti-vGlut2, AB2251-I, 1:1000, Millipore, USA) at 4°C overnight. On the following day, the sections were rinsed in KPBS containing 0.25% BSA and 0.1% Triton X-100 and incubated for 1 h in secondary antibody (Goat, Anti-guinea pig, Alex-568, A-11075, 1:400, ThermoFisher Scientific, USA). Following further rinsing, the sections were mounted with Fluoroshield (with DAPI, #F6057, SigmaAldrich), air-dried and cover slipped (size #0, Menzel Gläser). Fluorescent images were obtained with confocal laser scanning microscope (Leica LSM900).

## Results

Measurements of transcallosal LFPs were performed 4 weeks after surgery (*N* = 17) and 9–17 weeks after surgery (*N* = 8). Four of the animals were included in the both the early and late measurement. Five of the early and two of the eight late recordings had to be discarded due to noisy data or failure to elicit a transcallosal response with optogenetic stimulation. This leaves 18 recordings from 15 animals). Each animal is only represented once, even if two successful recording sessions were available. There was no immediate correlation in the evoked response (neither peak amplitude nor peak latency) to time since the viral injection (Supplementary Figure 1). We thus pooled the experimental data acquired in 15 sessions: nine sessions at young age (∼30 days after surgery), and six sessions at older age (∼60–120 days after surgery).

We assessed the optogenetically evoked, transcallosal responses in left M1 of the duration-intensity combinations of optogenetic stimulation in right M1. Given that the timepoint of stimulation in the time course of the anesthetic paradigm might affect the evoked responses (Supplementary Figures 2–4), the conditions were pseudo-randomly intermingled. No aberrant or seizure-related responses were detected as a result of our optogenetic stimulation paradigm during dexmedetomidine anesthesia (Bortel et al., 2020). The ability to reliably evoke a transcallosal response gradually increased with stimulation duration and intensity with the two variables having a synergistic effect (Figure 2, stimulation durations <1 ms not shown). High stimulation intensity (5 or 10 mW) reliably evoked a transcallosal N1-response already at very short stimulus durations of 0.5 or 1 ms in all 15 recording sessions (Figures 3B,C). In contrast, a reliable P1 response could only be evoked in 11 of the 15 recording sessions (Figures 3A,D) even at stimulus combinations with high stimulation intensity and long stimulation durations. To systematically characterize the stimulation-response relationship between right M1 stimulation and transcallosal evoked respond in left M1, in the following we therefore focus on the N1 peak as the output metric of interest.

We further examined the response properties of the N1-peak in those stimulation conditions in which optogenetic stimulation of right M1 elicited a reliable response in left M1 (Figure 4). N1-peak responses became less variable, as indexed by a lower coefficient of variation (CoV), when using a high stimulation intensity level and long stimulation duration (Figure 4A). This is underlined by the number of animal sessions with a CoV below a threshold of 1.0 for each individual condition (Figure 4B). The following median output metrics are only shown for conditions with a CoV below 1.0 present in at least 20% of the animals (*N* ≥ 3). There was however not a clear causal effect on the latency of the evoked response, when changing stimulation parameters (Supplementary Figure 5).

**FIGURE 5:**
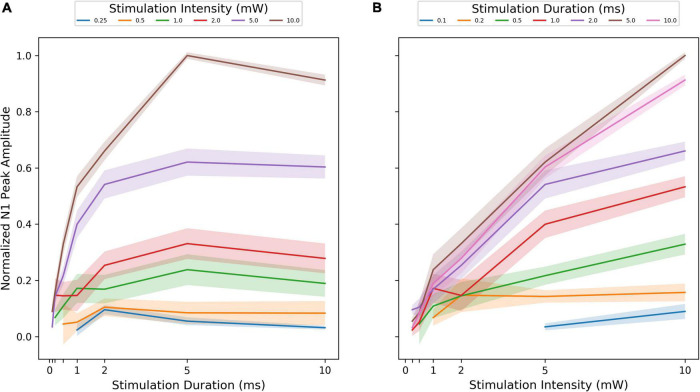
Input/Output-curves for all animals (*N* = 15). Y-axis reflects the N1 peak amplitude normalized by the maximum N1 amplitude of all conditions within a session for each animal. The solid line reflects the normalized group median value at a given condition, and the shadow around the line reflects the standard error of the median. **(A)** X-axis reflects the different stimulation durations (ms) and the different line colors reflect different stimulation intensities (mW). **(B)** X-axis reflects the different stimulation intensity (mW) and the different line colors reflect different stimulation durations (ms).

Regarding response magnitude, transcallosal evoked response gradually increased with the “dose” of optogenetic stimulation (Figure 4C). Our measurements revealed a statistically significant main effect of stimulation intensity on the normalized N1 peak amplitude (LMEM, *n* = 10,000, z = 13.11, *p* = 0.0001; Supplementary Figure 6), with a steady increase in amplitude with increasing stimulation intensity (Figures 4C, 5). Likewise, N1 peak amplitude was not significantly affected by stimulus duration (LMEM, *n* = 10,000, z = 1.74, *p* = 0.0392; Supplementary Figure 6), but with an apparent gradual increase until reaching a plateau at the longer durations of 5–10 ms (Figures 4C, 5). A significant interaction effect of stimulation duration and intensity support this synergy (LMEM, *n* = 10,000, z = 10.41, *p* = 0.0001; Supplementary Figure 6).

Depth of electrode and optic fiber position was measured from structural MR-scans in a random subset of the animals. Electrode depths were found to be 1,045 ± 24 μm (mean ± SEM; *N* = 5) and fiber depths were -1,139 ± 79 μm (mean ± SEM; *N* = 5) below the cortical surface.

## Discussion

We characterized the transcallosal cortical LFP response to optogenetic stimulation of excitatory neurons in the homologous contralateral M1 in rats. Systematic stimulation-response mapping revealed a gradual increase in the evoked transcallosal LFP response, when increasing the duration and intensity of laser stimulation—both individually and in combination. Conversely, the latency of the transcallosal M1 response were less affected by the stimulation parameters.

### Intracortical response evoked by transcallosal optogenetic stimulation

The cortical LFP responses elicited by optogenetic stimulation of excitatory transcallosal projections are in good agreement with previous optogenetic studies in rodents (Saiki et al., 2018; Böhm et al., 2020; Chen et al., 2020). During our experiments we did not detect any seizure-related responses due to optogenetic stimulation during dexmedetomidine anesthesia. In comparison with another study that did (Bortel et al., 2020), we used a slow-frequency optogenetic intracortical stimulation paradigm (∼1 Hz) whereas they experienced the aberrant responses at high-frequency (8 Hz) electric forepaw stimulation, but not at slower frequencies. We did not record long inter-stimulation periods as they did (∼90 s) during which the aberrant response arose in the experiments of Bortel and colleagues (Bortel et al., 2020), but we anticipate that seizures as long as they report (31.5 ± 1.83 ms) would have been visible from our single trial epochs.

Transcallosal interhemispheric signal conduction has also been probed with optogenetic stimulation of the somatosensory (Iordanova et al., 2018) and barrel cortex (Böhm et al., 2020; Chen et al., 2020). Iordanova et al. (2018) also targeted the excitatory neurons, but instead in the right somatosensory cortex and recorded the optogenetically evoked transcallosal response using silicon probes with linear electrode arrangement. This experimental set-up allowed them to compute the current source density throughout the cortical depth as a function of time after stimulus onset. Depth profiling of the LFP response showed that the polarity of the first LFP component flips with increasing depth of recording with an initial P1 peak emerging at deeper intracortical recording sites (Iordanova et al., 2018). Böhm et al. (2020) performed optogenetic stimulation above the barrel cortex in mice, and recorded epicranial responses in multiple areas. They reported similar evoked potentials with delays and amplitudes depending on the recording location, illustrating how the transcallosally evoked response spreads among cortical areas.

Electric stimulation in cats, monkeys and rats consistently showed interhemispheric responses that closely resembled the transcallosal responses evoked with cell-type specific optogenetic stimulation of excitatory projections in the present study (Curtis, 1940; Chang, 1953; Seggie and Berry, 1972; Mares et al., 1975; Innocenti et al., 1995). The CaMKIIa promoter has classically been and is often still associated with glutamatergic neurons and used target those neurons specifically (Liu and Jones, 1996; Aravanis et al., 2007; Basting et al., 2018; Iordanova et al., 2018). However, recently, the promoter has also been termed only indicative of excitatory neurons, as it might also be present in non-glutamatergic neurons (Lee et al., 2010; Just and Faber, 2019; Chen et al., 2020). It has been shown in rat, cat and mouse that some inhibitory GABAergic neurons also make transcallosal long-range connections with the contralateral cortex (Wahl and Ziemann, 2008; Rock et al., 2018). Electrical stimulation lacks cell-type specificity, and this should excite both glutamatergic and GABAergic transcallosal axons. The close resemblance of transcallosal potentials evoked by cell-type specific optogenetic stimulation and non-selective electrical stimulation suggests that the majority of transcallosal projections are indeed glutamatergic. Although we do show co-expression of vGlut2 and EYFP in (Supplementary Figures 7–9), a thorough validation that the evoked response is solely due to glutamatergic signaling was beyond the scope of the present study.

In our study, the most robust optogenetically evoked response was a negative peak (N1) in contralateral M1. A preceding positive peak (P1) was often detected, but only in approximately two thirds of the recording sessions. While we measured a quite consistent electrode depth (-1,045 ± 24 μm) across animals, even subtle differences in electrode locations can likely can be responsible for changed sensitivity to different configurations of the recorded LFPs (Iordanova et al., 2018). Our experimental approach was suited to reveal the magnitude of the transcallosal cortical response, but the recording procedures were not geared to identify which cortical neuronal populations were primarily responsive to transcallosal optogenetic stimulation. Since the transcallosally evoked potential (N1 or P1-N1 response) reflects the summated activity of neuronal populations located close to the recording electrode (Makarov et al., 2008; Buzsáki et al., 2012), we cannot infer which cell types or microcircuits were preferentially activated by the transcallosal glutamatergic input. Considering the existing literature (Conti and Manzoni, 1994; Wahl and Ziemann, 2008; Rock and Apicella, 2015), we hypothesize that the bulk of the cortical response reflects transsynaptically evoked intracortical inhibition and its secondary impact on intracortical neuronal activity. We therefore hypothesize that the initial brief P1 response might thus reflect the transcallosal excitation of GABAergic neurons that in turn govern the widespread long-lasting inhibition of activity, which may cause the N1 peak It is however also conceivable that the P1 includes transcallosal excitatory activation of excitatory circuits (Vanderwolf et al., 1987; Conti and Manzoni, 1994). Such conclusions however remain elusive with the methods used in this study and would among other things benefit from recordings from several depths in the cortical column.

However, cell-type or layer-specific activity readouts would be required to delineate the neurophysiology of the transcallosally evoked potential at the intracortical micro-circuit level (Kawaguchi, 1992; Hoffmeyer et al., 2007; Palmer et al., 2012, 2013). Furthermore, the administration of pulse pairs or high-frequency bursts may reveal the neuronal dynamics of optogenetically evoked, transcallosal M1-to-M1 interactions, similar to corresponding human TMS experiments (Daskalakis et al., 2002; Ni et al., 2020).

### Systematic evaluation of the optogenetic input parameters

The present study extends previous work on optogenetic stimulation of transcallosal projections in rodents. We systematically explored the impact of a various combinations of stimulus intensities and durations on the transcallosally evoked, cortical potential, covering the most commonly used stimulation variables (Aravanis et al., 2007; Huber et al., 2008; Lee et al., 2010; Desai et al., 2011; Christie et al., 2013; Kvitsiani et al., 2013; Iordanova et al., 2015; Yu et al., 2016; Shewcraft et al., 2020). We show that the probability to reliably evoke a transcallosal response in contralateral M1 gradually increased with the intensity and duration of the optogenetic stimulus. The transcallosally evoked N1 and P1 responses also became less variable and increased in magnitude at higher stimulus intensities and longer stimulus durations. This stimulus-response pattern mirrors the response pattern that can be induced with electrical stimulation (Chang, 1953), where the cortical neuronal response is determined by the relationship between the induced electrical current and the electrical properties of the neural tissue (Chang, 1953; Kuncel and Grill, 2004; Chaturvedi et al., 2013; Ramasubbu et al., 2018).

In the case of optogenetic stimulation, the efficacy to trigger axonal firing depends on how effectively the applied laser light activates light-sensitive ion channels in the neurons expressing channelrhodopsins (Hegemann et al., 2005; Grossman et al., 2011, 2013; Richter and Tan, 2014). Other studies have also demonstrated increasing magnitude of the optogenetically evoked response with increasing stimulation intensity and/or duration, *in vitro* (Hooks et al., 2015) and *in vivo* (Iordanova et al., 2018; Saiki et al., 2018; Böhm et al., 2020; Chen et al., 2020). These studies, however, did not perform a systematical mapping of the stimulation parameter space as in the present study. Taken together, our results show that it is possible to exploit the biophysics of optogenetic stimulation to probe the “gain function” of transcallosal M1-to-M1 excitatory projections in the rat brain. When interpreting the dose-response relationship as revealed by “optogenetic dose” and evoked transcallosal response magnitude, one needs to bear in mind that variations in dose will alter the area of effective stimulation. Optogenetic stimulation at higher intensity and longer duration may not only more effectively activate neurons located in the “hotspot” region of stimulation but also more effectively excite neurons in the brain tissue surrounding the hotspot, reducing anatomical specificity of the optogenetic intervention. While the optical properties of the implanted fiber determine the maximal range of optic stimulation, the area of effective stimulation will scale with stimulation intensity and duration within the range set by the fiber-based optoelectronics.

Since our examinations covered the lower range of intensity-duration combinations, the measurements also give indications regarding the minimal amplitude and duration needed to obtain a reliable transcallosal response (Figure 4). We can therefore conclude that intensities of 5–10 mW lasting 1–10 ms were sufficient in most animals to elicit a robust transcallosal response with our fiber-based optoelectronic set-up. Response magnitude increased with stimulation intensity without reaching a plateau, regardless of stimulation duration (Figures 4C, 5). Conversely, response magnitude did reach a plateau at a stimulation duration of 5 ms. Hence, the intensity parameters used in this experiment did not appear to cover the entire range of possible dose-response relationships. Based on the existing knowledge of synaptic delays and our data we can speculate why stimulation duration saturated, but stimulation intensity did not (Figure 5). Due to highly recurrent local connectivity in cortex, the average synaptic travel time from one stimulated neuron to its unstimulated neighbor and back to it would be below the duration of the stimulation duration. Therefore, increasing stimulation duration may not change the transcallosal LFP responses because the same cycle is being repeated. Other effects might occur in the cortex of the recording site after prolonged stimulation, such as shunting inhibition, reducing the response in the transcallosally activated cortical microcircuits (Paulus and Rothwell, 2016).

Stimulation intensity, on the other hand, has a chance to recruit weakly connected neurons and manifest itself as a change in transcallosal LFPs. High stimulation intensity may however also cause heating of the tissue, which introduces spurious findings in functional MRI (Christie et al., 2013) and may result in tissue damage (Cardin et al., 2010; Han, 2012; Dai et al., 2015; Gysbrechts et al., 2016; Senova et al., 2017). Further, it may also illuminate an unreasonably large tissue volume, hampering spatial specificity (Saiki et al., 2018). Indeed, care should be taken to restrict the stimulation volume to the transfected neuron population in the brain region of interest (Aravanis et al., 2007). This could principally be handled by simulations of the light transmission within the tissue, as has been carried out by others (Aravanis et al., 2007; Williams et al., 2013; Luboeinski and Tchumatchenko, 2020). Such simulations should however be accompanied by careful titration of the viral inoculum and histological validation. However, to make realistic predictions on the effects of stimulus intensity and duration on the ChR2 expressing neuron ensemble, would require building a neural network model. Such a model should include inhibitory and excitatory cell-types, biologically plausible synaptic connectivity matrices and with intrinsic biophysical properties of different cell-types in different layers. Without such a model, we limit ourselves to recommending to restrict stimulus intensity and duration to the low-to-moderate dose range, when studying dose-response relationships with optogenetic stimulation.

In electrical stimulation studies, a short stimulus duration is usually desired, to minimize the duration of the electrical artifact and to better depict the direct result of the stimulation. The former concern is less of an issue in optogenetic studies, as there are no immediate stimulation artifacts, although photo-electric artifacts have been reported when using optrodes (Cardin et al., 2010; Park et al., 2014). But an unnecessarily long stimulation duration may complicate the physiological interpretation of the optogenetically evoked cortical response.

### Implications and limitations

Our LFP results highlight the importance to characterize the dose-response relationship in optogenetic stimulation studies. Detailed knowledge about the dose-response properties is critical to the selection of the optimal stimulation parameters and to understanding the evoked neuronal response. A recent study measured locally evoked spiking and LFP activity at the site of cortical optogenetic stimulation in awake, alert non-human primates confirms our conclusion (Shewcraft et al., 2020). In that study, the selection of stimulation variables determined the stimulation-evoked, excitatory-inhibitory responses, highlighting the role of in-depth understanding of dose-response relationships in optogenetic stimulation studies.

Our results also form a solid basis for future studies on the functional integrity of the motor transcallosal fibers in rodents. Our approach allows to reliably investigate functional aspects of a specific cortico-cortical interhemispheric pathway. A next step will be to add non-invasive microstructural mapping techniques to relate the dose-response relationships at the functional level with the microstructural properties of the transcallosal fiber tract, including *post mortem* histological validation (Dyrby et al., 2018; Andersson et al., 2020, 2022). Further, this preclinical platform can be used translationally to assess animal disease models (e.g., multiple sclerosis, stroke, trauma) that affect the transcallosal M1–M1 pathway. This will open novel possibilities to study the function-structure relationship of the transcallosal motor pathway and how it is affected by disease.

In two rats we were able to acquire two similar recording sessions (data not shown), obtained approximately eight weeks apart. The introduction of the closed implant protector for the latest animals included in this study, would likely result in fewer damages and losses of the fiber implant and electrode pedestal, and allow for more subsequent recordings of the same animals. This possibility to repeatedly probe directional functional connectivity between the right and left M1 opens for longitudinal investigations of e.g., how transcallosal motor pathways are functionally shaped by maturation, aging, specific brain diseases, or experimental manipulations (i.e., training, pharmacological challenges).

### Methodological considerations

The study has some methodological limitations. Electrophysiological recordings and analyses focused on single-electrode data and population responses as reflected by the LFP. The use of multi-electrode arrays covering different depth levels of the cortex and analysis of single-unit activity would have yielded a more complete picture of the transcallosally evoked cortical response pattern with respect to cortical layers (Voigt et al., 2017).

With the viral injection strategy used in this study, we would not have been able to reach and transfect neurons from only one layer. To target the callosally projecting neurons specifically could have been achieved using a more advanced injection strategy—for instance with dependent viral expression (Lima et al., 2009; Yizhar et al., 2011; Senn et al., 2014), using e.g., Cre-recombinase (Nagy, 2000; Hnasko et al., 2006).

Our approach also was not able to disentangle the contribution of various cell types, e.g., pyramidal cells and inhibitory interneurons, to the transcallosally evoked LFP. Pharmacological manipulations or the use of cell-specific readouts of functional activity may be used in future studies to dissect the various components of the transcallosal response at the micro-circuit level. A similar consideration applies to optogenetic stimulation. The use of more cell-type specific viral vectors might help to reveal which cortical cell types are the main contributors to the transcallosal interaction and how they can be most effectively stimulated and modulated with interventional neurostimulation. We used AAV serotype 5 aiming for anterograde viral transfection. We can however not exclude that callosal neurons projecting from the contralateral hemisphere might have taken the viral construct up retrogradely (Taymans et al., 2007; McFarland et al., 2009; Mason et al., 2010; Aschauer et al., 2013; Rook et al., 2021), which in turn potentially could result in antidromic contamination of the evoked LFPs. Additionally, AAV-5 have been shown to produce no transsynaptic anterograde viral trafficking (Zingg et al., 2017), which could otherwise have resulted in symmetric back-projection onto the injected and stimulated hemisphere. Finally, our histological investigations did not reveal any somatic EYFP expression in the contralateral hemisphere, indicating that the transfection at least primarily have occurred anterogradely, producing a minimal risk of contamination with antidromic action potentials.

All recordings were performed under general anesthesia to have a setup where the brain state is stable. This naturally limits the translation of the results to the physiological states present in normal wakeful behaving animals. Our anesthesia protocol was optimized for future concurrent use with MRI (Pan et al., 2013; Magnuson et al., 2014) which will enable us to investigate structural and functional transcallosal connectivity using several modalities.

## Conclusion

By systematically mapping the relationship between “stimulation dose” and “response magnitude,” we were able to characterize the gain function of transcallosal M1-to-M1 excitatory connections in the rat brain, using a set-up that is suited for long-term *in vivo* stimulation and recordings. Combining unilateral optogenetic stimulation of excitatory neurons with intracortical recordings of the transcallosally evoked response has substantial translational potential to foster a better understanding of functional M1–M1 transcallosal interactions and to inform interventions that use transcranial cortex stimulation to modify the balance in transcallosal M1–M1 interaction.

The observed dose-response profiles of the N1 peak in terms of variability and magnitude suggest that one should aim at finding the optimal trade-off between stimulation duration and intensity in optogenetic studies. Although evoked responses become more robust at higher stimulation intensities and longer stimulation durations, other intensity-duration combinations may be preferable depending on the research question. In many cases, the intensity-duration combination needs to be sufficiently strong to elicit a robust transcallosal response, but not too strong to secure sufficient sensitivity toward dynamic changes in transcallosal connectivity that are experimentally induced or arise across the lifespan.

## Data availability statement

The raw data supporting the conclusions of this article will be made available by the authors, without undue reservation.

## Ethics statement

The animal study was reviewed and approved by the Animal Experiments Inspectorate (2016-15-0201-00868), Denmark.

## Author contributions

CSS, TBD, and HRS contributed to the conception of the project idea and project planning. CSS carried out the surgeries, established the experimental setup, performed experiments, data analysis, and statistics, and wrote the first draft of the manuscript. LT, TBD, DK, and HRS provided valuable inputs to the data analysis. CSS, LT, DK, TBD, and HRS interpreted the results. All authors contributed to and critically revised the final manuscript and have approved the content for publication and agree to be accountable for all aspects of the work in ensuring that questions related to the accuracy or integrity of any part of the work are appropriately investigated and resolved.

## Footnotes

1
https://git.drcmr.dk/cskoven/lab

2
https://git.drcmr.dk/cskoven/nonin

3
https://git.drcmr.dk/cskoven/elphys

4
https://github.com/open-ephys/analysistools
