# Supplementary material for: Dose-response relationship between the variables of unilateral optogenetic stimulation and transcallosal evoked responses in rat motor cortex
Source: Front Neurosci. 2022 Sep 23;16:968839. doi: 10.3389/fnins.2022.968839 (PMC9539969; doi:10.3389/fnins.2022.968839)
Supplement: Supplementary file 1 [file Table_1.DOCX]

Supplementary Material

**a)**

**b)**


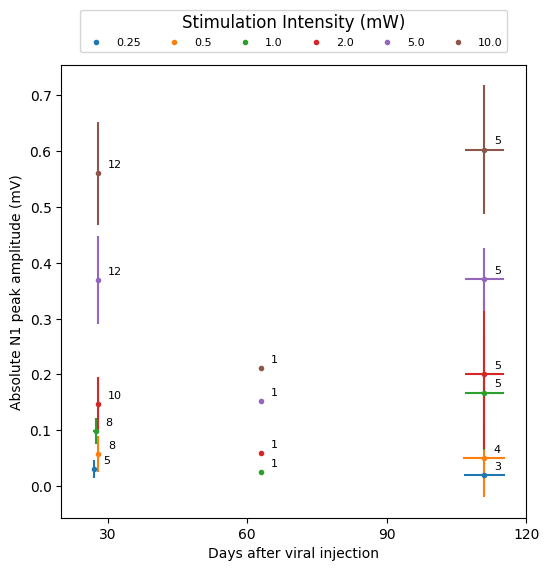

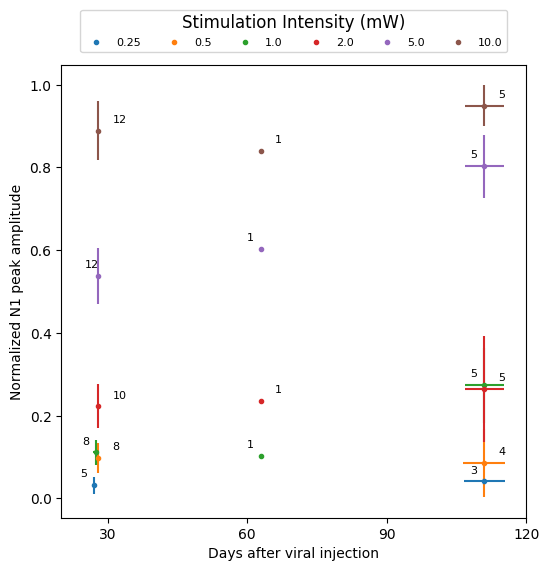


**c)**

**d)**


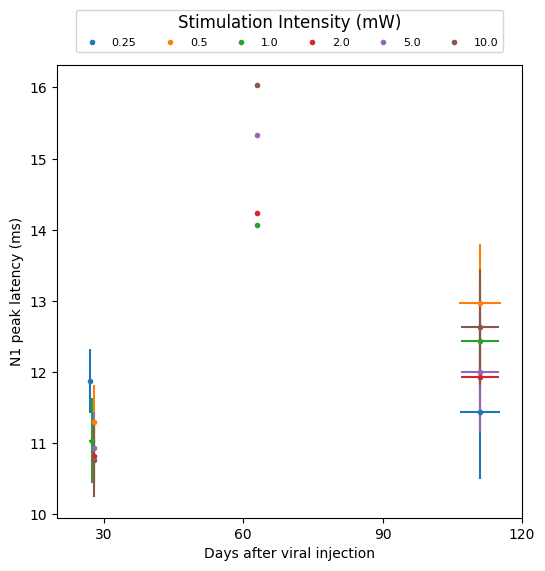

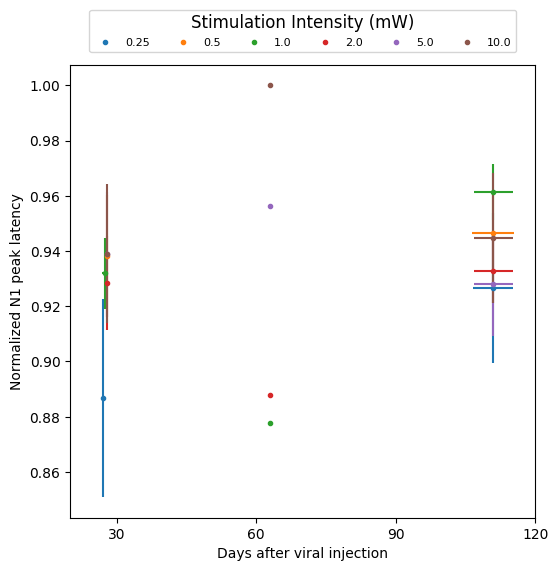


**Suppl. Fig. 1:** Cross-sectional illustration of the **(a)** absolute N1 peak amplitude **(b)** normalized N1 peak amplitude **(c)** absolute N1 peak latency and **(d)** normalized N1 peak latency – as a function of days after viral injection. The stimulation duration is fixed (10 ms) and the 6 different stimulation intensities are represented by colors. Animal recording sessions are grouped in three pools (“young”: 27-32 days [N=12]; “medium age”: 63 days [N=1] and “old”: 98-120 [N=5]). Three animals occur both in the young and old groups. Horizontal lines represent SEM of the median recording day for each group. Vertical lines represent SEM around the normalized amplitude for the given condition (stimulation intensity) for all animal sessions in the group.


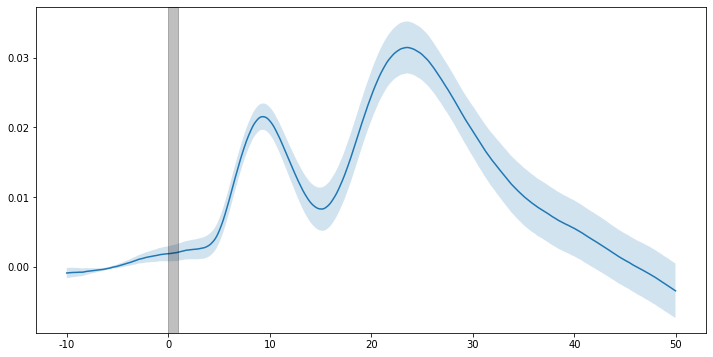


**Suppl. Fig. 2:** Average trace (mean of all paradigms) for 1 animal (rat35.3) after stimulation with 1.0 ms (illustrated by vertical grey rectangle) at 2.0 mW. Solid blue line: mean; light blue shadow: SEM.


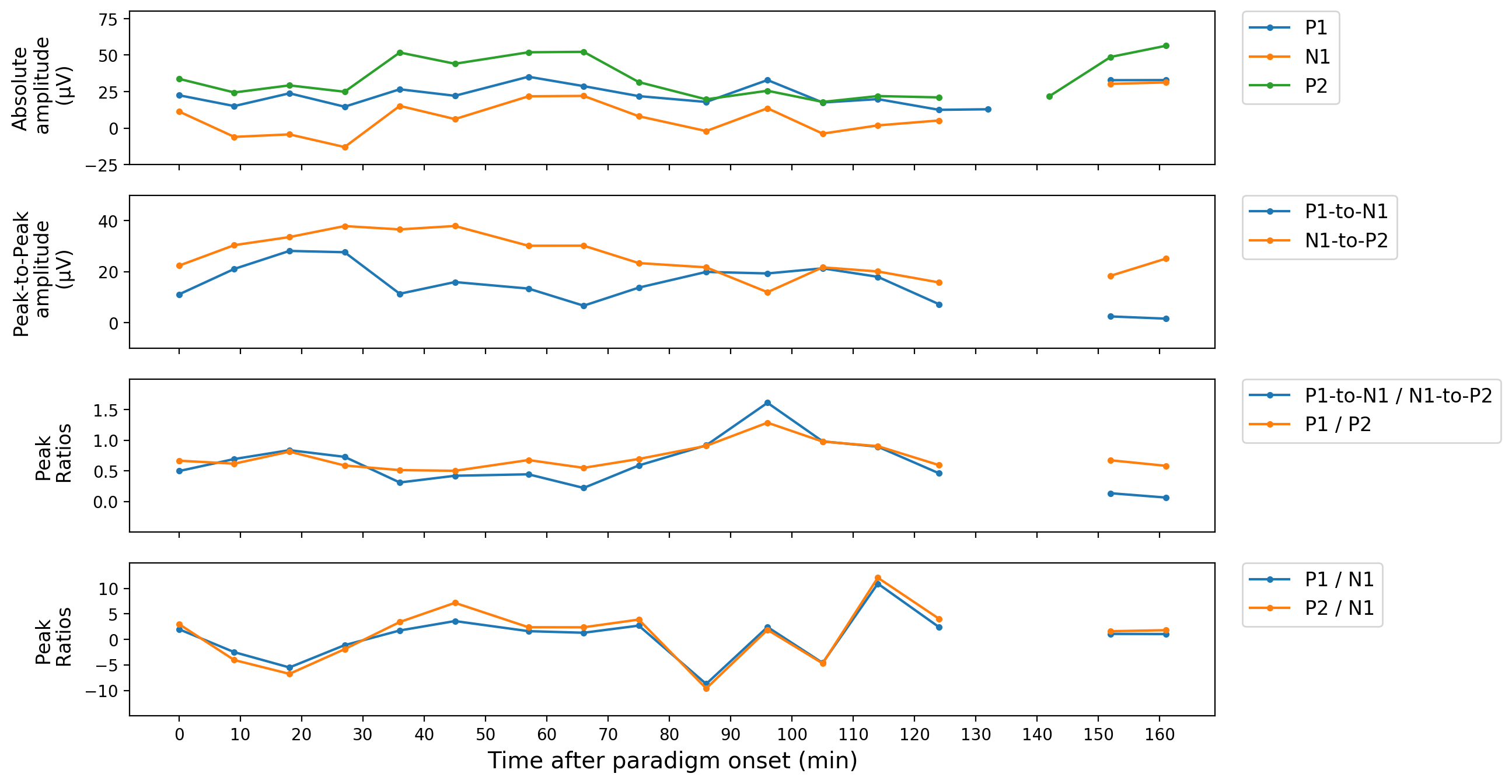


**Suppl. Fig. 3:** Corresponding time courses of peak amplitudes peak amplitudes and ratios for a single animal (rat35.3), with constant stimulation parameters (1.0 ms @ 2.0 mW). **Panel 1:** Absolute amplitude of P1 (blue), N1 (orange) and P2 (green), as a function of time since paradigm onset. **Panel 2:** Peak-to-peak amplitude values of P1-N1 (blue) and N1-P2 (orange). **Panel 3:** Peak ratios of the peak-to-peak values of P1-N1/N1-P2 (blue) and ratios of absolute peak amplitudes of P1/P2 (orange). **Panel 4:** Peak ratios of absolute values of P1/N1 (blue) and P2/N1.


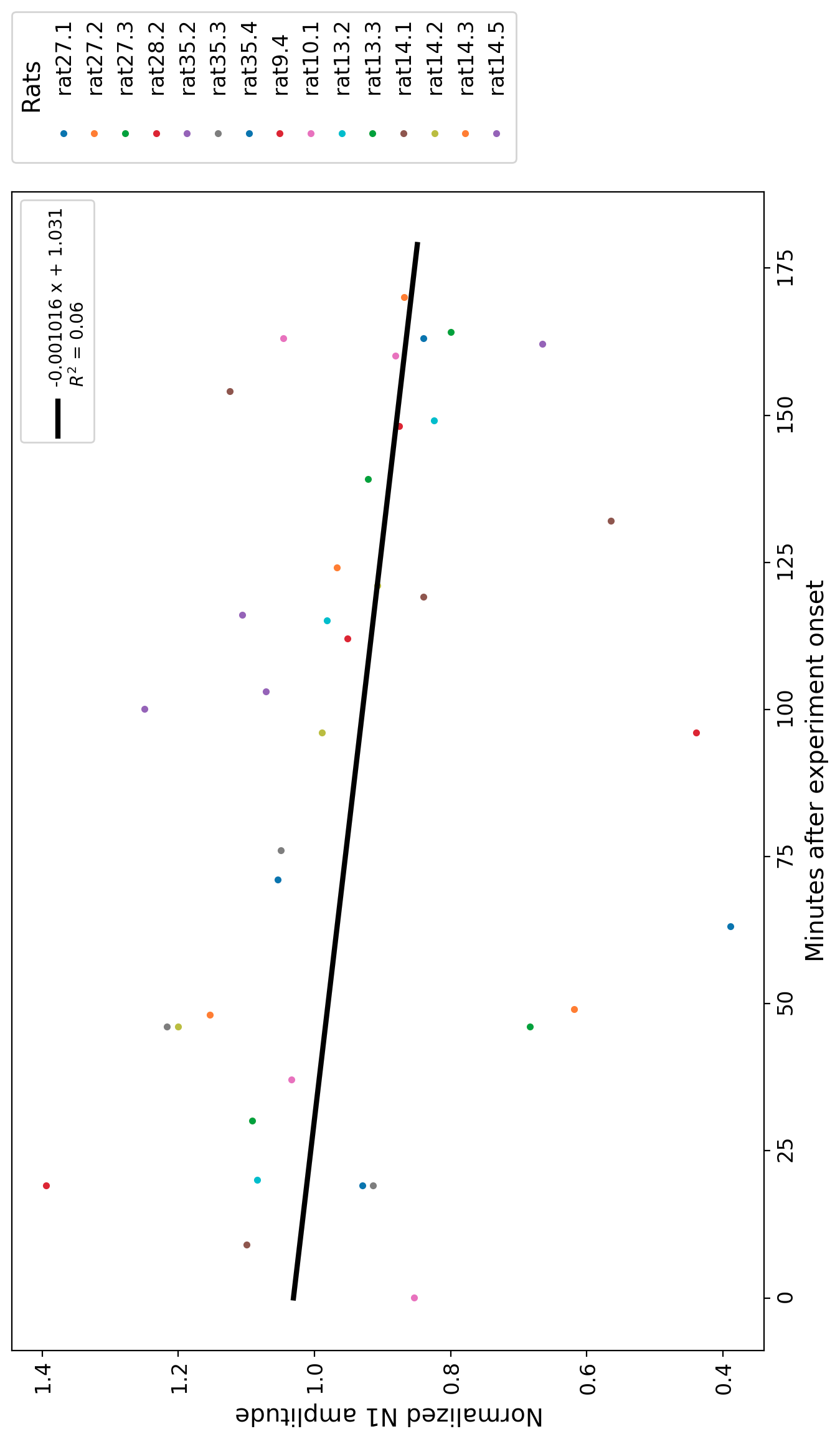


**Suppl. Fig. 4:** Cross-sectional time course of the normalized N1 peak amplitude for a selected stimulation condition (10 ms @ 10 mW). The blocks corresponding to the chosen condition are placed randomly across the whole parameter mapping experiment time course, for each animal. The N1 peak amplitude is normalized to the maximum mean peak amplitude in the entire session (of all conditions). The peak amplitudes from each block (eg. 50 trials) are normalized with respect to the maximum amplitude of all condition averages (150 trials per condition). Therefore, results above 1.0 could be achieved. The timepoint of the condition block within the experiment session (minutes after experiment onset) is reflected on the x-axis.


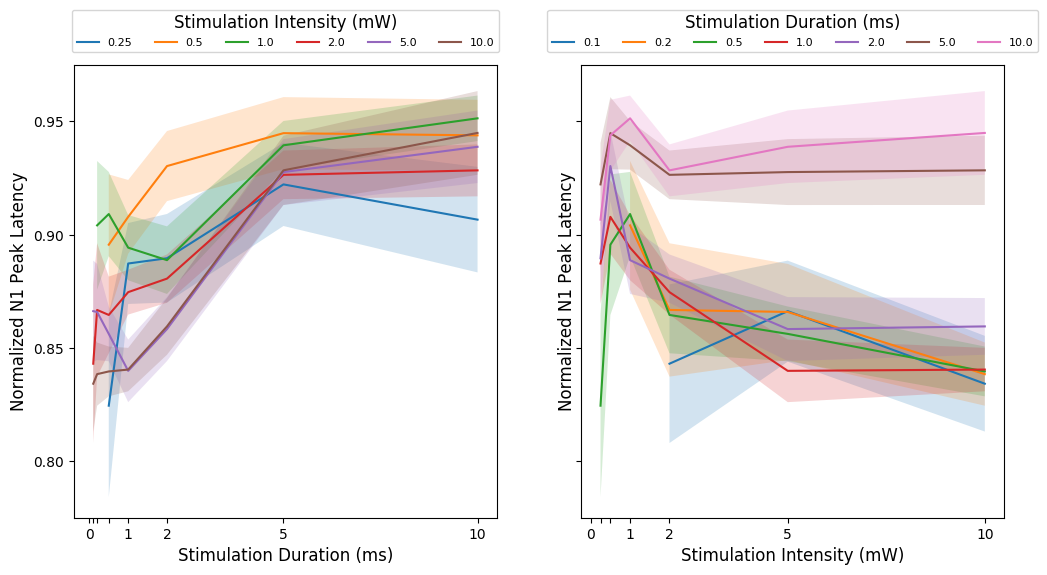


**Suppl.Fig. 5:** Input/Latency-curves for all animals (N=15). Y-axis reflects the N1 peak latency normalized by the longest N1 peak latency of all conditions within a session for each animal. The solid line reflects the normalized group median latency at a given condition, and the shadow around the line reflects the standard error of the median. **Left panel:** X-axis reflects the different stimulation durations (ms) and the different line colors reflect different stimulation intensities (mW). **Right panel:** X-axis reflects the different stimulation intensity (mW) and the different line colors reflect different stimulation durations (ms).


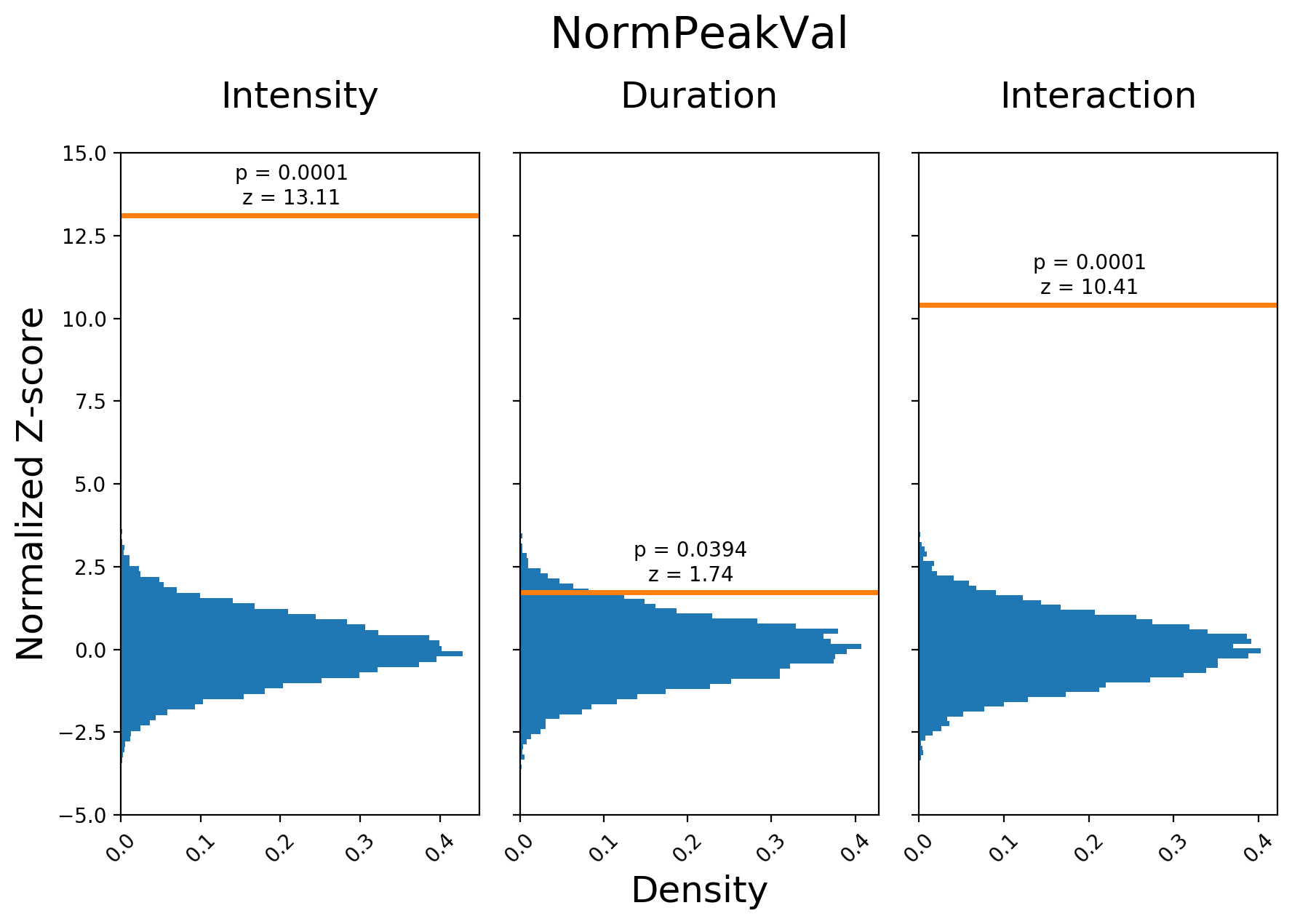


**Suppl. Fig. 6:** Permutation test of mixed effects model (n=10000 per effect) of normalized N1 peak amplitude, with stimulation intensity and duration as fixed effects and the subject ID as a random effect. The normalized N1 peak amplitude thus appear significantly affected by stimulation intensity (p=0.0001, z=13.11) as well as by an interaction of stimulation intensity and duration (p=0.0001, z=10.41) – and principally also by stimulation duration (p=0.0394, z=1.74). All z-scores are normalized with respect to distribution mean and standard deviation.


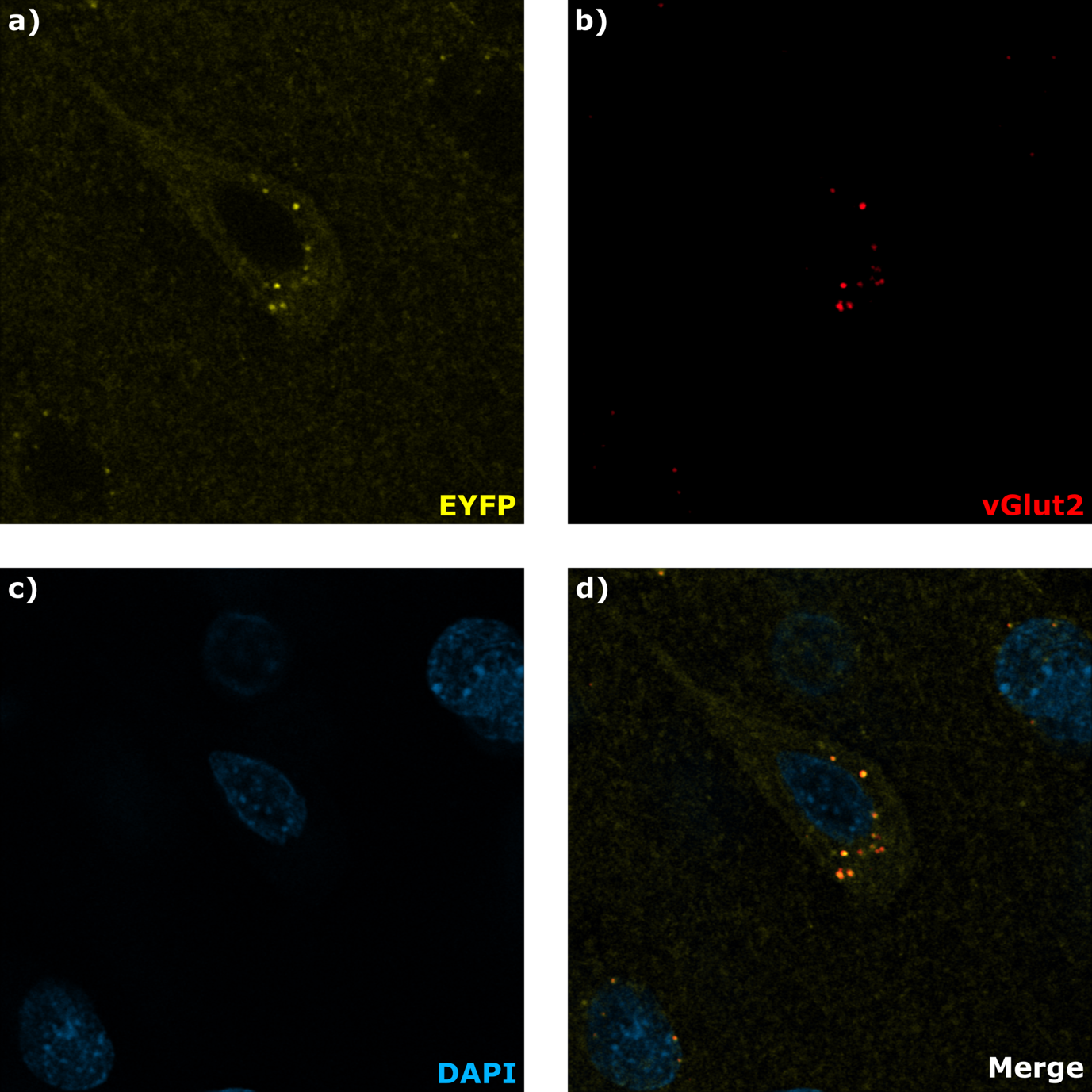


**Suppl. Fig. 7:** Representative images from confocal microscopy (10x) in right M1 of 2021_rat6.1 illustrating: **(a)** an EYFP-positive neuron (yellow), **(b)** vGlut2 expression (red) and **(c)** DAPI-stain (blue) indicating cell nuclei, and **(d)** a composite image, indicating that the EYFP-positive neuron also has vGlut2.


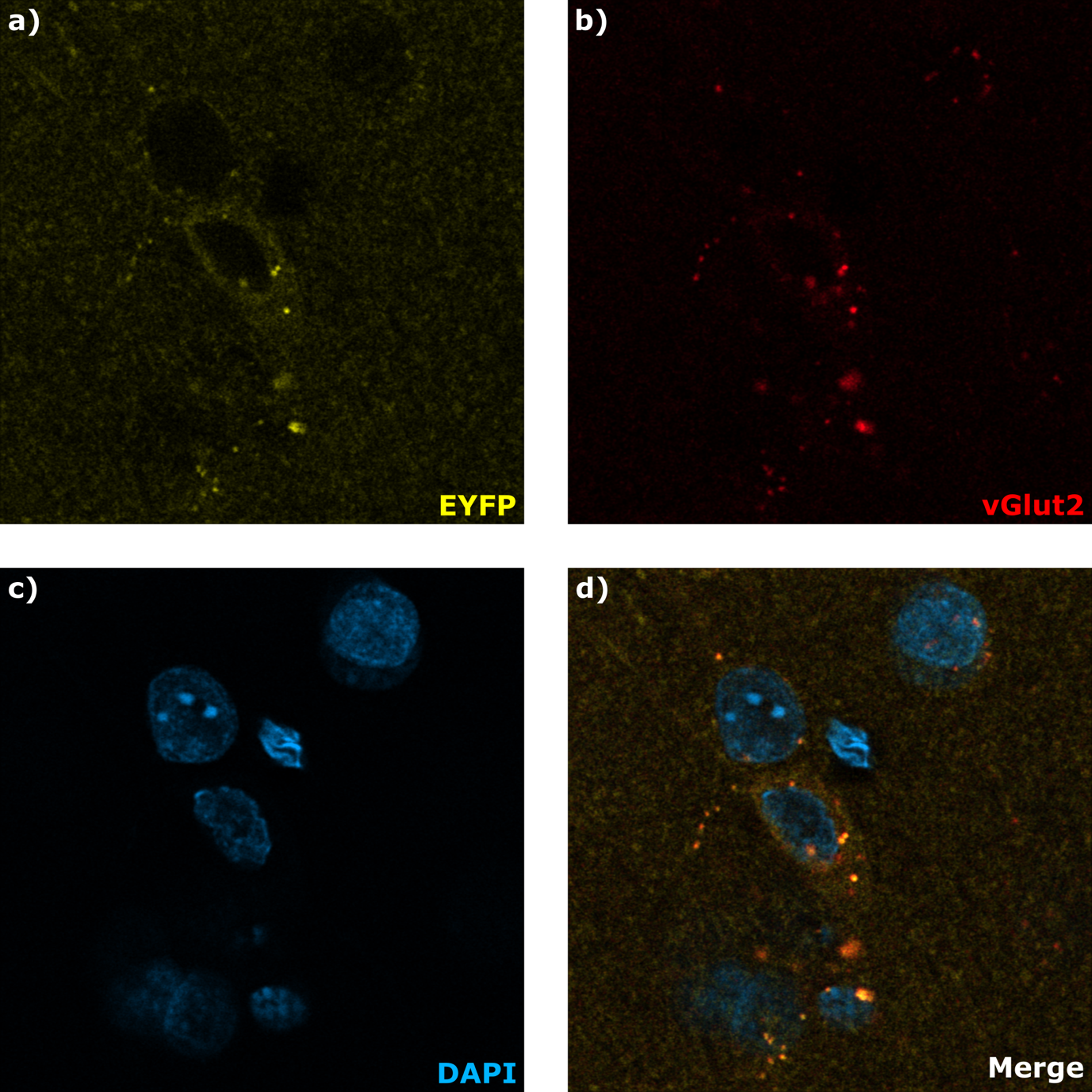


**Suppl. Fig. 8:** Another set of representative images (similar to Suppl. Fig 6) from confocal microscopy (10x) in right M1 of 2021_rat6.1 illustrating: **(a)** an EYFP-positive neuron (yellow) **(b)** vGlut2 expression (red) and **(c)** DAPI-stain (blue) indicating cell nuclei, and **(d)** a composite image, indicating that the EYFP-positive neuron also has vGlut2.


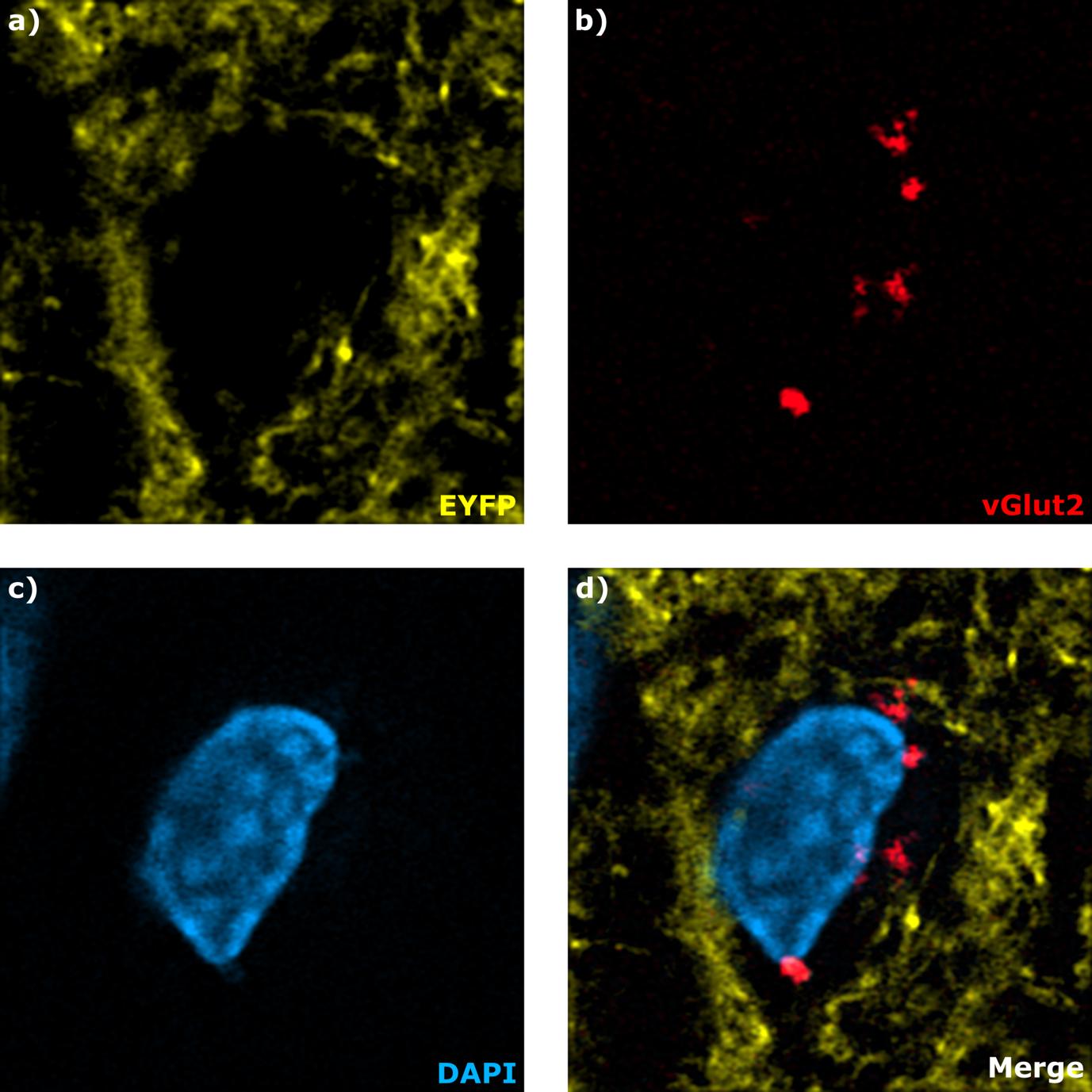


**Suppl. Fig. 9:** Representative image from confocal microscopy (40x) in right M1 of 2021_rat6.2 illustrating: **(a)** EYFP- expression (yellow), **(b)** vGlut2 expression (red) and **(c)** DAPI-stain (blue) indicating cell nuclei, and **(d)** a composite image.
